# Exogenous putrescine enhances salt tolerance in *Populus nigra* × *maximowiczii*: growth, physiological, and biochemical responses

**DOI:** 10.3389/fpls.2025.1641288

**Published:** 2026-02-27

**Authors:** Sanchari Kundu, Medini Weerasinghe, Maegan Gagne, Subhash Minocha

**Affiliations:** Department of Biological Science, University of New Hampshire, Durham, NH, United States

**Keywords:** amino acids, foliar spray, growth, hybrid poplar, metabolism, polyamines, putrescine, salt stress

## Abstract

**Introduction:**

Putrescine, a polyamine involved in plant growth and stress responses, has shown potential in mitigating abiotic stress effects. However, little is known about the effects of exogenous addition of putrescine regarding salt tolerance in trees.

**Methods:**

This study was conducted to investigate whether exogenous putrescine application via foliar spray enhances growth in a hybrid poplar (*Populus nigra x maximowiczii*, clone NM6) under a short duration of salt stress. Salt stress was induced by irrigating roots with 100 mM and 200 mM NaCl, followed by foliar spraying of putrescine on several days. Measurement of growth including plant height and stem diameter for each plant were recorded in the greenhouse every 15 days throughout the experiment. Gas exchange, total chlorophyll, carotenoids, soluble sugars and proteins, amino acids, polyamines, and relative water content were analyzed in foliage collected 3, 6, 7, 13, 20, 35 days after treatment.

**Results:**

Foliar putrescine application significantly promoted growth, increasing stem height by ~20% and stem diameter by ~15% under 100 mM NaCl compared to untreated plants. Foliar spray significantly enhanced fructose accumulation, with ~37% higher levels at day 6 under 100 mM NaCl compared to unsprayed plants and increased sucrose by ~28% at day 13. Based on metabolic responses, plants treated with 100 mM NaCl fared better when sprayed with putrescine than those treated with 200 mM NaCl.

**Discussion:**

Exogenous application of putrescine alleviated salt-induced growth inhibition, likely through its role in maintaining osmotic balance and energy metabolism. These findings highlight the potential of exogenous putrescine treatment as a practical strategy to enhance salt tolerance in young poplar trees, with implications for forestry and land reclamation in saline environments.

## 1 Introduction

Polyamines (PAs), such as putrescine (Put), spermidine (Spd), and spermine (Spm), are small aliphatic amines derived from amino acids (AAs) that play essential roles in plant development and adaptation to abiotic stress (Alcázar et al., 2006; Minocha et al., 2014; Pál et al., 2018; Atabayeva et al., 2020; Antoniou et al., 2021; Shao et al., 2022; Borromeo et al., 2023; Blázquez, 2024; Tabur et al., 2024; Zhao et al., 2024b). They influence diverse physiological processes such as seed germination, photosynthesis, root architecture, and cellular signaling. In recent years, PAs have attracted attention for their involvement in enhancing stress tolerance through metabolic and physiological modulation (Wuddineh et al., 2017; Antoniou et al., 2021; Imran et al., 2021; Sundararajan et al., 2021; Borromeo et al., 2023; Pál et al., 2025). However, their role in woody plants, particularly fast-growing poplars, remains unexplored despite their significant ecological and economic importance (Zalesny et al., 2019). Long life cycles, complex anatomical structures, and ethical considerations in genetic studies, all create unique challenges in research with woody plants (Neale and Kremer, 2011; Chen et al., 2021). Understanding the stress adaptation mechanisms in woody perennial plants is crucial for sustainable forestry and climate resilience, particularly in response to global warming.

Among various abiotic stresses, salinity is a major factor that disrupts plant growth, photosynthesis, ion homeostasis, water balance, and, carbon and nitrogen metabolism (Bargmann et al., 2009; Cramer et al., 2011; Dinneny, 2015; Parihar et al., 2015; Hanson et al., 2016; Haak et al., 2017; Zhang et al., 2020a; Sun et al., 2022; Calhoun et al., 2023; Kundu, 2023; Zhang et al., 2023). Azimi et al. (2021) reported that N deficiency is a major consequence of salt stress, reducing leaf area and inducing chlorosis, which hinders plant productivity. While N fertilization is a common approach for mitigation, run-off from excessive application of N can contribute to environmental issues such as water pollution and algal blooms, necessitating more feasible stress mitigation strategies (Li et al., 2021; Zhang et al., 2020a).

PAs have been recognized as key regulators of abiotic stress responses, accumulating in plants subjected to salinity, heavy metals, drought, and temperature extremes (Alcázar et al., 2010; Mohapatra et al., 2010; Pál et al., 2018; Malik et al., 2022; Kundu, 2023; Wang et al., 2024b; Liu et al., 2025b). They play a vital role in modulating C metabolism, stabilizing membrane integrity, scavenging reactive oxygen species (ROS), and regulating osmotic balance (Majumdar et al., 2013; Pál et al., 2021, 2025). PAs can also enhance N assimilation, thereby improving chlorophyll synthesis and overall plant growth (Alcázar et al., 2010; Tamang et al., 2021; Zhang et al., 2024). Several studies have demonstrated that exogenous PA application improves plant resilience by modulating hormone signaling like abscisic acid, gene expression, and enzymatic responses under salt stress and others (Paul et al., 2018; Xiong et al., 2018; Borromeo et al., 2023; Wang et al., 2024b). Seed priming with PAs has been reported to enhance photosynthetic pigments, proline accumulation, root growth and biomass production in crops such as soybean, rapeseed, tomato, and rice (Sheteiwy et al., 2021; ElSayed et al., 2022; Borromeo et al., 2023; Zhang et al., 2024) under salinity stress. Additionally, exogenous PA treatments have been shown to increase soluble sugars, proteins, and amino acid accumulation, enhancing abiotic stress tolerance in economically significant plants like grapevine, ginseng, rice, and tomato (Sundararajan et al., 2021). Recent studies have also shown that exogenous Spd treatment increased resistance to fusariosis in flax by suppression of PA metabolism (Augustyniak et al., 2025). However, despite these promising findings, studies in woody perennials remain limited, particularly in fast-growing trees like poplars.

Poplars (*Populus* spp.) are among the fastest-growing perennial tree species, widely distributed across North America, China and India (Dickmann, 2001; Hawkins et al., 2003; Plomion et al., 2016; Zalesny et al., 2019; Buell et al., 2023); and are key resources used in sustainable forestry, bioenergy production, and the lumber industry (Yi et al., 2022). Poplars exhibit notable stress tolerance, including resistance to salt and heavy metal contamination (Zalesny et al., 2019; Lin et al., 2023), making them ideal candidates for genetic and physiological research (Guerra et al., 2011). Several varieties of poplars have shown potential to be grown in marginal sandy lands (Ghezehei et al., 2021). Advances in omics technologies and the sequencing of the poplar genome (Ma et al., 2019) have enabled deeper insights into their stress response pathways. Notably, transgenic studies in hybrid poplars have identified genes like *NAC13*, which enable salt tolerance (Zhang et al., 2019). Some recent studies have applied single-cell RNA-seq transcriptomics (scRNA-seq) to dissect poplar vascular root system and adaptations in response to soil compaction and abiotic stress, revealing cell-type-specific regulatory mechanisms (Conde et al., 2022; Liu et al., 2025a; Li et al., 2024; Zhu et al., 2025). Recent computational advances have further enabled cross-species comparison of conserved cell types, expanding the potential for identifying PA-mediated functions across plant taxa at single-cell resolution (Chau et al., 2025). Such approaches offer promising avenues for future research on PA specific stress responses in woody perennials. Understanding how PAs contribute to salt stress tolerance at tissue- or cell-specific resolution could bridge a critical knowledge gap in tree physiology and enhance strategies for improving stress resilience in forest trees.

Among poplar hybrids, *Populus nigra x maximowiczii* (NM6 clone) is widely cultivated in North America due to its fast growth, high biomass yield, asexual propagation capacity, and genetic stability (Labrecque and Teodorescu, 2005; Guerra et al., 2011). Zalesny et al. (2019) also reported that the NM6 clone was more salt-tolerant than other poplar varieties. Yet no studies have investigated the physiological and biochemical effects of exogenous PA application in mitigating salt stress in NM6 poplars. Given the increasing soil salinity issues affecting forestry and agricultural productivity, understanding how PA treatments effect stress tolerance in NM6 poplars is of scientific as well as economic significance.

To address this research gap, we investigated the role of exogenous PA application (specifically Put) in enhancing salt stress tolerance in young NM6 poplars. In a controlled greenhouse experiment, we assessed the physiological and biochemical responses of NM6 poplar cuttings to NaCl-induced stress and examined whether foliar-application of Put could alleviate its adverse effects. The objective of this study was to determine how exogenous application of Put influences plant responses to salt stress, with a focus on physiological traits, photosynthetic performance, and key metabolic indicators. To guide this investigation, we formulated the following research questions: 1. How does NaCl stress affect the physiological and metabolic processes in NM6 poplar plants? 2. Can the exogenous application of Put mitigate NaCl-induced stress; and if so, through which biochemical and physiological pathways? We hypothesized that foliar application of Put would enhance salt stress tolerance in NM6 poplars by improving plant growth, photosynthesis, and metabolic stability (soluble sugars, AAs, and PAs) thereby promoting growth and stress resilience. This study contributes novel insights into PA-mediated stress mitigation in woody plants and advances sustainable strategies to improve abiotic stress in forestry species.

## 2 Materials and methods

### 2.1 Plant material and growth conditions

Cuttings of hybrid poplar (*Populus nigra x maximowiczii* - NM6) plant were collected from a 5-year-old, healthy tree at the University of New Hampshire (UNH) Kingman Farm in Durham (43.1725143, -70.9462316). The cuttings were rooted and maintained at the UNH MacFarlane greenhouse from mid-April to late June 2021 as an acclimatization period prior to the start of experimental treatments in early July.

During the initial growth phase, cuttings (~15 cm in height, 0.6 cm stem diameter) were kept under mist for two weeks in 17.78 cm grow tubes containing PRO-MIX^®^ soil with Mycorrhizae peat based growing medium (Premier Tech, Quebec, Cananda). Each 17.78 cm grow tube (approximately 5 cm in diameter) was filled with an estimated 349 cm³ of PRO-MIX^®^ soil with Mycorrhizae. The grow tubes were placed in trays to retain excess water, ensuring consistent soil moisture, and plants were watered regularly. After 2 months, well-established plants (~30–38 cm in height with 9–10 leaves) were selected and transferred to 33 cm pots filled with a 1:1 mixture of vermiculite and perlite.

The experiments were conducted from July to September 2021 in a controlled greenhouse environment with natural sunlight and a 16-hour photoperiod. The temperature in the greenhouse ranged between 22°C and 24°C, with a relative humidity of 70%. Plants were irrigated and fertilized twice daily using an automated drip-line irrigation system with Jack’s Pure Water LX - Professional fertilizer^®^ (J.R. Peters Inc., Allentown, PA, USA). Each plant received 200 mL of water at 08:00 AM and 2:00 PM, and additional watering with plain water was applied manually in the evening when needed.

### 2.2 Experimental design

As the plants were large (33-cm pots; ~3-month-old canopies) and greenhouse space and irrigation manifolds were limited, we used a fixed-position, nonrandomized block design. One time salt treatment with sodium chloride (NaCl) was added 21 days after transplanting the cuttings into 33 cm pots, when the plants were approximately 3 months after the initial collection of cuttings. There was a total of 36 cuttings, 6 treatment assigned to 6 treatment groups (n = 6 per treatment): treatment A (control- no NaCl, no Put), treatment B (100 mM NaCl), treatment C (200 mM NaCl), treatment D (control + 1 mM Put foliar spray), treatment E (100 mM NaCl + 1 mM Put foliar spray), and treatment F (200 mM NaCl + 1 mM Put foliar spray).

A 0.5% Silwet™ (Momentive Performance Materials, Niskayuna, NY, USA) solution was added as a surfactant to enhance the penetration of the 1 mM Put (dihydrochloride ≥ 98%; Sigma-Aldrich, Inc., St. Louis, MO, USA) foliar spray. To ensure uniform salt application, drip irrigation was halted 18 hours before salt treatment. Salt treatments were delivered via root irrigation by hand-pouring 200 mL of NaCl solution per pot at the time zero of treatment (Day 0) and again 6 hours later. Drip irrigation resumed 6 hours after the final salt application. Saucers were placed beneath the pots to prevent water leakage.

Thirty milliliters of 1 mM Put foliar spray was applied to cuttings in treatment groups D, E, and F immediately following the salt treatment. The spray was applied only to the leaves while the soil was carefully covered with plastic sheets to prevent contamination. After spraying, any excess solution was manually tapped off the leaves. Put spray occurred at time 0, and days 3, 6, and 13. Workflow for the experimental design is added in **Supplementary Figure 1**.

### 2.3 Sample collection

Plant samples including the remaining leaves and root tissues, were collected at various time points before and after treatment application for physiological and biochemical analyses.

#### 2.3.1 Leaf sampling

All cuttings were sampled at time zero and one representative plant per treatment group was sampled 3, 6, 13, and 20 days post treatment. For each sampling, the fully expanded 6^th^ leaf from the plant apex was selected, washed with fresh water and patted dry between 2 layers of paper towels. From those leaves ~6 mm disks were punched using a common paper punch to create a pool of ~2 g. The leaf disks were mixed, and sub-samples were taken for various analyses; each placed into pre-weighed 2 ml microfuge tubes and appropriate volumes and buffers added. On the day of collection, all samples were collected and transported on ice and later stored at -20°C until further analysis.

Physiological traits like relative water content (RWC) and chlorophyll content were measured using leaf discs. Gas exchange measurements were taken with LICOR-6400 directly on the plant leaves. Biochemical assays like soluble sugars, PAs, AAs, and total protein were done with leaf discs. All samples (except leaf discs for biomass measurements) were stored at -20°C until further analyses.

#### 2.3.2 Roots sampling

Root samples were collected from 4 biological replicates per treatment only once at the time of harvesting. Roots were thoroughly washed under running tap water to remove residual vermiculite and perlite and pat-dried. Fine secondary roots (2–3 mm in diameter) were carefully snipped from the main root using sterilized scissors. A pool of ~50 mg of fresh root tissue was collected, mixed thoroughly, places into a pre-weighed 2 ml microfuge tubes and 1 ml 5% perchloric acid (HCLO_4_) was added. All root samples were collected and transported on ice and later stored at -20°C until further analysis.

### 2.4 Plant growth parameters

Plant height and stem diameter measurements were taken from 6 replicates per treatment and recorded at time 0 and on days 15, 30, and 45 post salt treatment. Basal diameters were measured using a digital caliper, with a consistent reference point marked near the base of the stem to ensure accuracy across measurement intervals. Growth in height and stem diameter was expressed as a percentage increase using the following formulas:


$$\begin{array}{c} \text{Increase in height }\left(\%\right)=[\left(\text{Height on next day}-\text{height on previous day}\right)/\\ \text{Height of day }1]*100 \end{array}$$

$$\begin{array}{c} \text{Increase in diameter }\left(\%\right)=\left[\text{Diameter on next day}–\text{diameter on previous day}\right)/\\ \text{Diameter of day }1]*100 \end{array}$$

### 2.5 Soluble sugars

*Sample preparation:* Soluble sugars were quantified following a modified protocol from Blagden et al. (2022). Leaf discs (50 ± 2 mg FW) were incubated at 65 °C for 30 min in 1 mL of 80% ethanol.

*Analysis:* A total of 11 sugars were quantified: xylose, arabinose, fructose, mannose, glucose, galactose, sucrose, trehalose, rhamnose monohydrate, maltose monohydrate, and raffinose pentahydrate. Detection was performed using a Shimadzu RID-10A refractive index detector (RID) set at 30°C (Shimadzu Scientific Instruments Inc., Columbia, MD). The total run time was 15 min, including column washing and stabilization between injections. The column temperature was maintained at 25°C throughout the analysis. Chromatographic data were processed using Perkin Elmer TotalChrom software (version 6.2.1). Peaks were identified by matching retention times with known sugar standards. An 8-point external standard curve (3 mg ml^−1^) was generated for individual sugar quantification (**Supplementary Table 2**). Unresolved sugar peaks were quantified as combined concentrations based on their summed peak areas, including xylose + arabinose, glucose + galactose, and trehalose + maltose.

### 2.6 Polyamines and amino acids

*Sample preparation:* the quantification of different AAs and PAs, approximately 40 ± 2 mg fresh leaf discs were collected in 5% HCLO_4_ at a ratio of 1:25 (w:v) in 2 ml microfuge tubes. The samples were freeze-thawed three times and processed for dansylation following a modified protocol from Minocha et al. (1994), (Minocha and Long, 2004) and (McDermot et al., 2020). After the final thawing, the samples were vortexed at high speed for 2 min and centrifuged at 14,000 × g for 8 min. For each sample and external standards, 20 µl of an internal standards mix containing 0.05 mM α-methyl-DL phenylalanine (for AAs) and 0.05 mm heptane diamine (for PAs), both dissolved in 5% HCLO_4_, was added to each tube. Subsequently, 100 µl of the freeze-thawed extract was combined with 100 µl of 2.691 M sodium carbonate and 100 µl of freshly prepared dansyl chloride (20 mg/mL in acetone). The mixture was vortexed and incubated at 60°C for 30 min. After incubation, the microfuge tubes were cooled at room temperature for 3 min, followed by the addition of 45 µl glacial acetic acid to terminate the reaction. Acetone was evaporated using a speed-vac for 10 min, and 1735 µl methanol was added to the mixture. The methanol extract was filtered using a 0.45 µm nylon syringe filter fitted onto a 3 ml syringe before transferring the solution into autosampler vials.

*Analysis:* Separation of AAs and PAs was performed using a 15 cm column (Phenomenex Synergi Hydro-RP 80 Å, LC Column 150 x 4.6 mm, 4 µm). Quantification was achieved using a fluorescence detector (Series 200 PerkinElmer) set at 340 nm for excitation and 510 nm for emission. Relative quantification of AAs and PAs was performed using an external standard curve (**Supplementary Table 3**). Chromatographic data were analyzed using Perkin Elmer TotalChrom software (version 6.2.1), incorporating a multiplication factor within the software to determine the concentration of each component in nmol g^−1^ FW of tissue.

### 2.7 Total soluble proteins

Total soluble protein content was quantified following a modified protocol from Minocha et al. (2019). Fresh leaf discs (50 ± 2 mg FW) were extracted in a freshly prepared 100 mM Tris buffer (pH 8.0) containing 20 mM MgCl_2_, 10 mM NaHCO_3_, 1 mM ethylenediaminetetraacetic acid (EDTA), and 10% (v/v) glycerol. The extraction was performed using a three-cycle freeze-thaw method.

The extract was centrifuged at 13,000 × g for 5 min, and the supernatant was used for total soluble protein quantification following the Bradford (1976) method. The analysis was conducted using Bio-Rad protein assay dye reagent (Bio-Rad Laboratories, Hercules, CA), with bovine serum albumin (BSA) as the standard. Absorbance was recorded at 595 nm using a spectrophotometer. A standard curve (0.1–0.5 mg/ml) was generated to quantify protein concentrations in the samples.

### 2.8 Relative water content

Relative water content (RWC) was determined by measuring the fresh weight (FW) of the leaf tissue immediately after collection. For DW, the leaf samples were incubated in an oven at 70°C for 48 hours. The samples were reweighed after 24 hours to confirm that no residual moisture remained. FW/DW ratios were also recorded and are presented alongside the RWC data to provide a complementary measure of tissue hydration status. RWC was calculated using the following formula:


RWC (%)=[(FW-DW)/FW]*100

### 2.9 Chlorophyll content

Chlorophyll content was quantified following the method described in Minocha et al. (2009) and McDermot et al. (2020). Total chlorophyll, chlorophyll a, chlorophyll b, and carotenoids were calculated using equations found in Lichtenthaler (1987).

### 2.10 Leaf gas exchange

Gas exchange parameters, including photosynthetic rate (P_n_), transpiration rate (E), and stomatal conductance (g_s_) were measured on the 6^th^ fully expanded leaf from the apex for each cutting. Measurements were taken on 3–4 plants per treatment using a portable Li-COR photosynthesis system (LI-6800/XT, Li-COR Biosciences, Lincoln, NE, USA).

Gas exchange was recorded between 09:00 and 12:00 under a photosynthetic photon flux density (PPFD) of 1000 μmol m^−2^ s^−1^ provided by a red-blue LED light source (6 cm² chamber). Airflow was maintained at 500 μmol s^−1^, and reference CO_2_ concentrations were set to 400 μmol mol^−1^. The block temperature was maintained at 25°C. Leaf humidity was not actively controlled but ranged between 50–60% during measurements.

### 2.11 Statistical analysis

All statistical analyses were conducted with JMP^®^ Pro 18 (JMP Statistical Discovery LLC, Cary, NC, USA). Data were analyzed using a two-way analysis of variance (ANOVA) to assess statistically significant differences between treatments within each time point. All results are presented using the original, untransformed data. Time and treatment were treated as fixed effects. *Post-hoc* comparisons were performed using Tukey’s test, with statistical significance set at p< 0.05. Graphical figures of the data were generated using the ggplot2 package, and both heatmap and correlation plots were produced with the pheatmap and corrplot packages in RStudio (R version 4.4.1; Posit, Boston, MA, USA).

## 3 Results

### 3.1 Effects of put spray on plant growth and morphological symptoms

**Figure 1:**
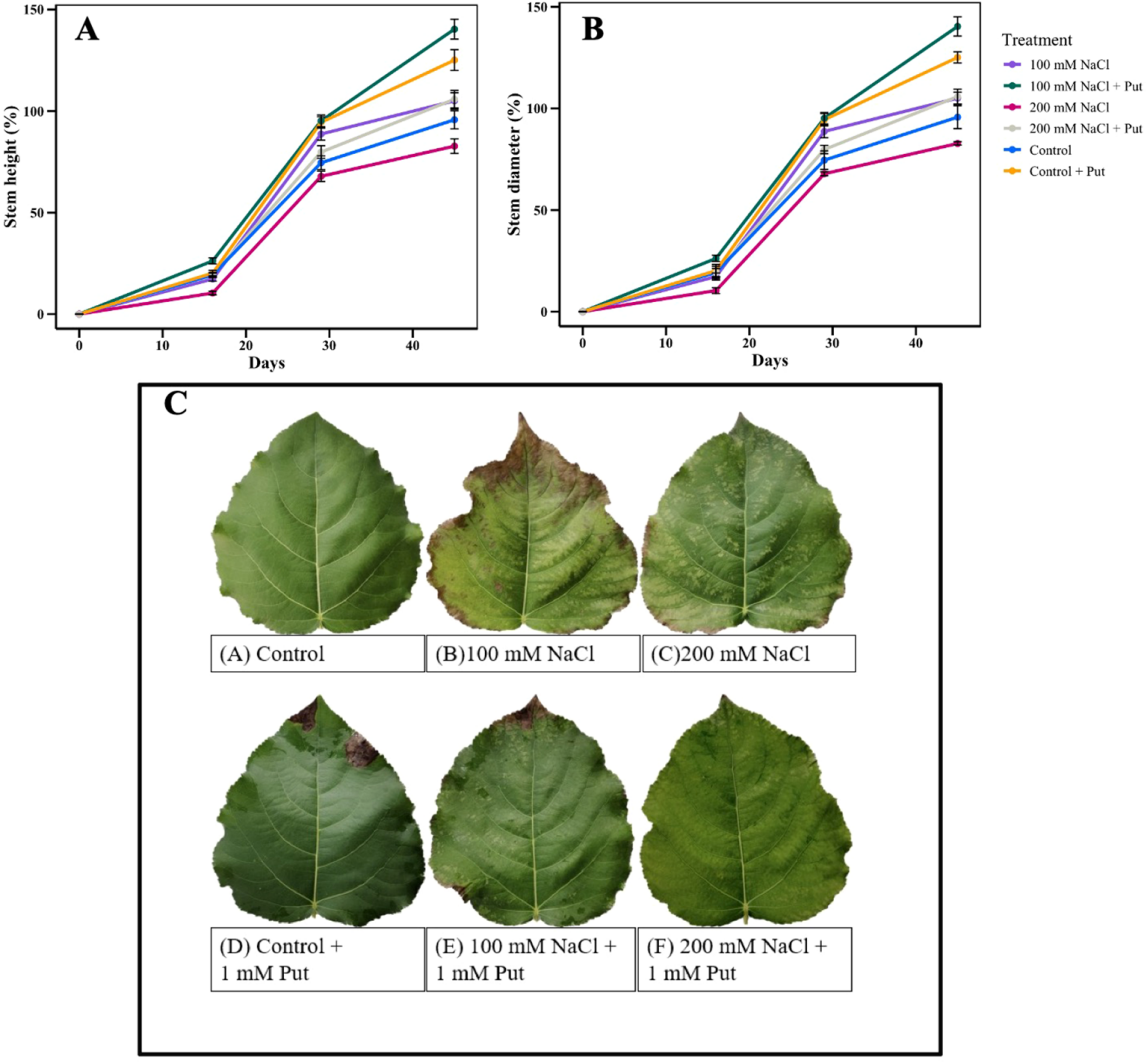
The effect of two different concentrations of NaCl (± Putrescine spray) on the growth and morphology of hybrid poplar NM6 plants. **(A, B)** Average stem length and stem diameter changes over 45 days of salt treatment. **(C)** Morphological symptoms of leaves under different treatments: Control, 100 mM NaCl, 200 mM NaCl, Control + 1 mM Put, 100 mM NaCl + 1 mM Put, 200 mM NaCl + 1 mM Put. Data represent mean ± SE (n = 6).

Plant height and stem diameter were measured 15, 30 and 45 days after salt treatment. **Figures 1B, C** shows that both stem length and diameter in the control and 100 mM NaCl treated cuttings sprayed with foliar Put are greater than either of those treatments without Put spray. At 45 days, Put increased stem height by approximately 20–25% and stem diameter by 15–18% under 100 mM NaCl compared to untreated plants. That suggests that under control or low salt conditions, Put had a growth-promoting effect on the poplar cuttings. Foliar discoloration (**Figure 1A**) began 13 days after salt exposure; leaves of the 100 mM and 200 mM NaCl treated cuttings appear to have a greater degree of chlorosis and necrosis as compared to the control. Application of 1 mM Put reduced these stress symptoms in the salt-treated plants, and all Put-treated leaves displayed a healthier appearance, with markedly less chlorosis and/or necrosis.

### 3.2 Effects of put spray on gas exchange, chlorophyll and total soluble protein

**Figure 2:**
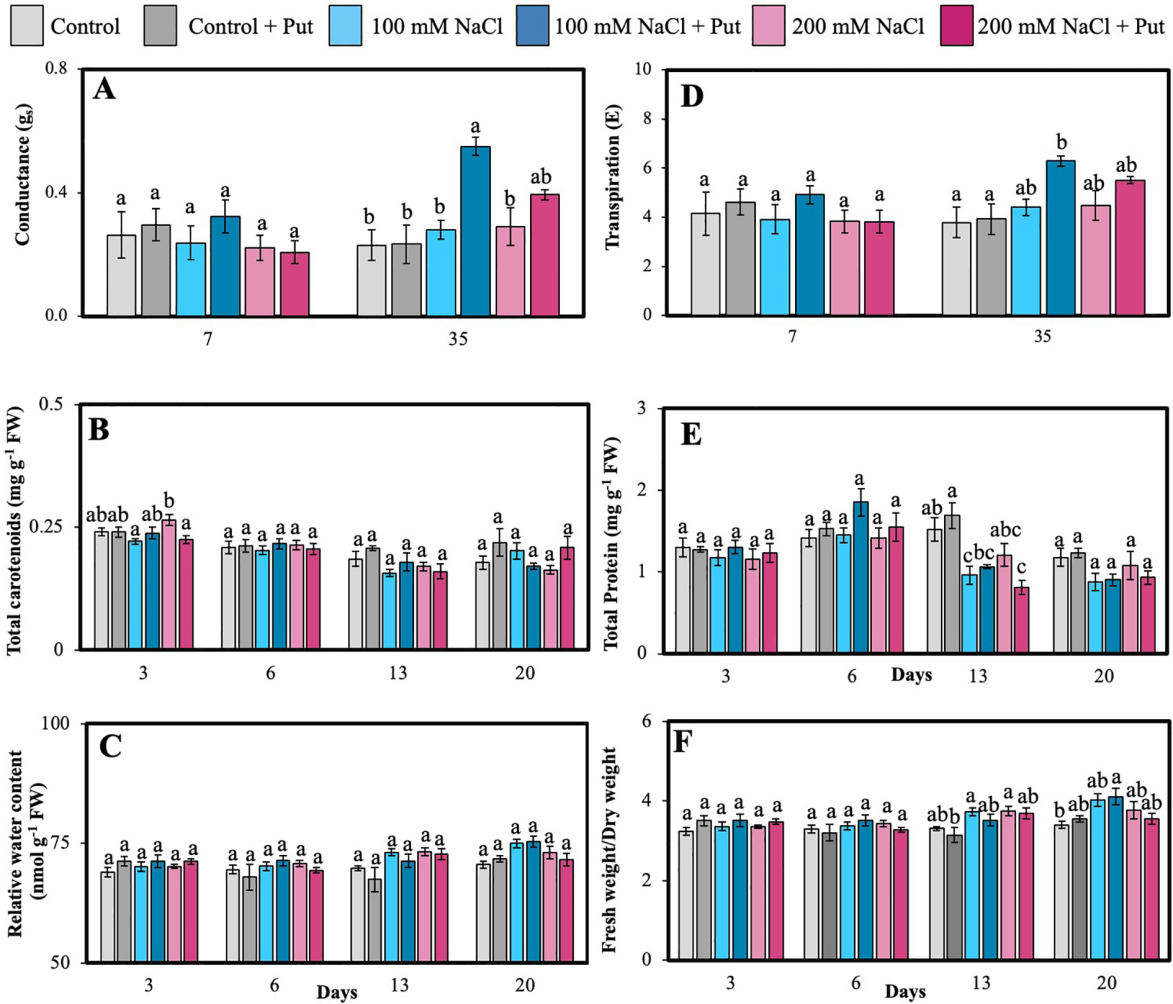
Effect of two different concentrations of NaCl (± Putrescine spray) on physiological accumulation in hybrid poplar NM6 leaves over time. **(A)** Conductance, **(B)** Carotenoid, **(C)** Relative water content, **(D)** Transpiration, **(E)** Protein, and **(F)** Fresh weight/dry weight measured at several days after salt treatment. Different letters indicate statistically significant differences (p< 0.05) among treatments. Data represent mean ± SE (n = 3, 5).

Gas exchange parameters, carotenoid levels, and protein content were measured in hybrid poplar NM6 leaves across multiple time points after NaCl and Put treatment. g_s_ was significantly affected by treatment on day 35. (**Figure 2A**). On day 35, g_s_ in the Put-treated 100 mM NaCl plants reached 0.55± 0.03 mol m^−2^ s^−1^, which was significantly higher than in the control (0.230 ± 0.053 mol m^−2^ s^−1^; p< 0.05). On day 7, no significant differences were observed among treatments. *E* showed similar trends (**Figure 2D**). On day 35, plants treated with 100 mM NaCl + Put had significantly higher *E* (~6.29 ± 0.2 mmol m^−2^ s^−1^) compared to the control (~5.1 ± 0.3 mmol m^−2^ s^−1^) and 100 mM NaCl alone (~3.79 ± 0.61 mmol m^−2^ s^−1^; *p* < 0.05). *Pn* was 62% higher.

(however, non-significant) in Put-treated plants under both 100 mM and 200 mM NaCl compared to untreated salt-stressed groups (see **Supplementary Figure 2C**).

Total carotenoids (**Figure 2B**) peaked on day 3 under 200 mM NaCl + Put, reaching 0.39 ± 0.01 mg g^−1^ FW, which was significantly higher than 200 mM NaCl alone (0.31 ± 0.01 mg g^−1^ FW, *p* < 0.05). Total soluble protein content (**Figure 2E**) dropped under salt stress, with the 100 mM NaCl group reaching a low of 2.0 ± 0.08 mg g^−1^ FW on day 13, significantly less than the control (2.6 ± 0.07 mg g^−1^ FW, *p* < 0.05). Put treatment partially restored protein levels to 2.3 ± 0.09 mg g^−1^ FW, indicating a moderate recovery.

RWC (**Figure 2C**) remained stable across treatments and time points, ranging between 71–75%, with no significant differences detected (**Figure 2C**). The ratio of FW/DW (**Figure 2F**) was significantly higher in 100 mM NaCl-treated plants on day 13 (3.6 ± 0.15) compared to Put-sprayed control plants (2.9 ± 0.12, *p* < 0.05), possibly indicating osmotic adjustment.

### 3.3 Effects of put *s*pray on soluble sugar content

**Figure 3:**
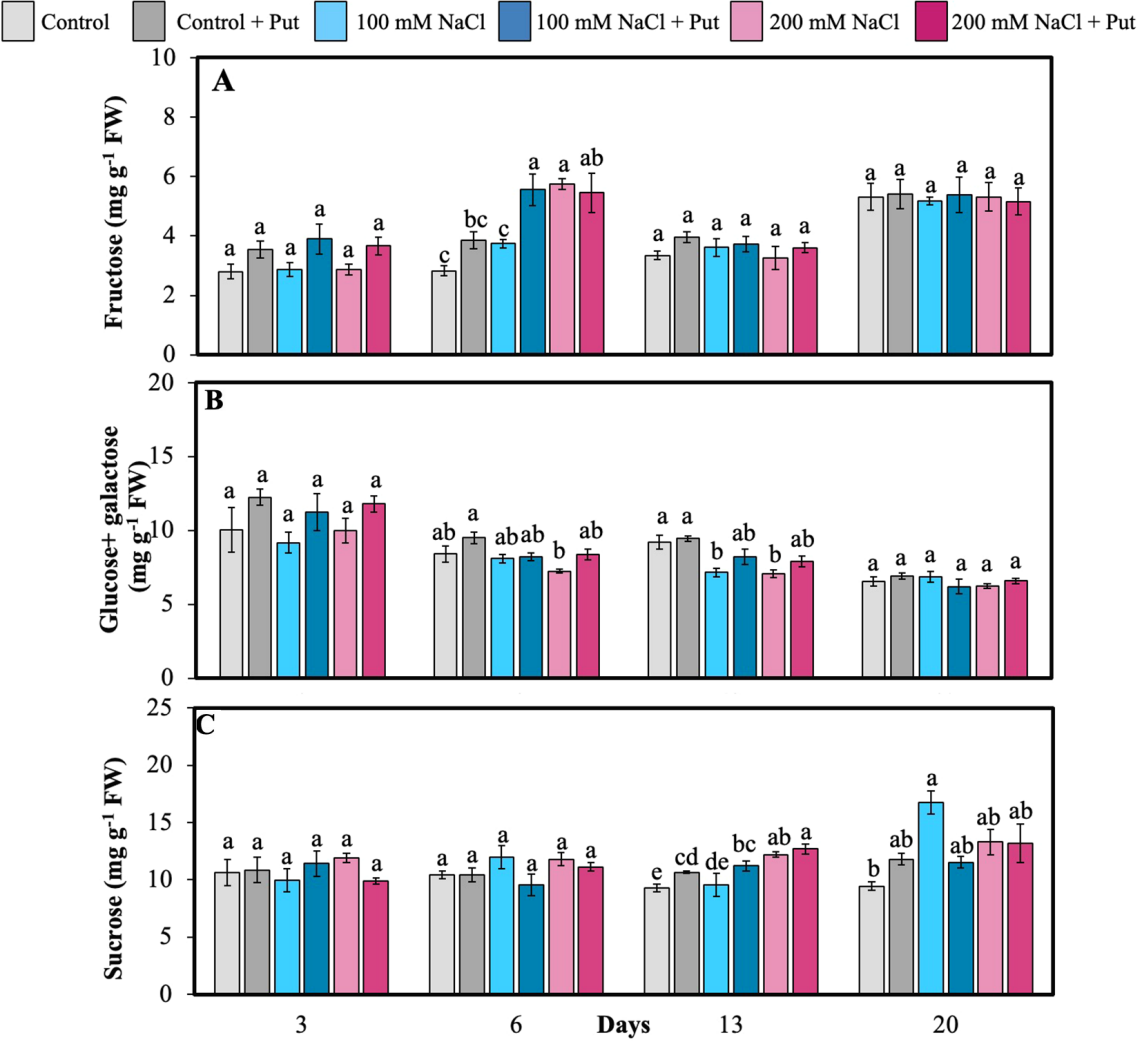
Effect of two different concentrations of NaCl (± Putrescine spray) on soluble sugar accumulation in hybrid poplar NM6 leaves over time. **(A)** Fructose, **(B)** Glucose + Galactose, and **(C)** Sucrose measured at 3, 6, 13, and 20 days after salt treatment. Different letters indicate statistically significant differences (p< 0.05) among treatments. Data represent mean ± SE (n = 5).

Soluble sugar (fructose, glucose + galactose, and sucrose) was quantified in the 6^th^ fully matured leaf at multiple time points following NaCl and Put treatment. Fructose content significantly increased under Put-sprayed 100 mM NaCl *vs.* unsprayed at 6^th^ day (**Figure 3A**). Fructose was significantly higher in 200 mM NaCl treated plants compared to control and 100 mM NaCl treated plants on the 6^th^ day. Glucose and galactose were non-separable in our HPLC system and hence they are represented together. Glucose + galactose levels were significantly lower in 200 mM NaCl treated plants compared to Put sprayed control plants on the 6^th^ and 13^th^ day (**Figure 3B**). Sucrose levels were significantly lower in Put-sprayed plants *vs.* unsprayed for 100 mM NaCl on the 6^th^ day (**Figure 3C**). Sucrose was significantly higher for Put-sprayed 200 mM NaCl plants on the 13^th^ day. Sucrose was also significantly higher in Put-sprayed plants than those unsprayed for control and 100 mM NaCl on the 13^th^ day. Our results also showed that sucrose contents were also significantly higher in 100 mM NaCl treated plants compared to the control plants on 20^th^ day.

The overall temporal shift in sucrose levels may reflect stress-induced C partitioning, wherein sucrose is either broken down or synthesized depending on the phase and intensity of salt stress. Early reductions (e.g., day 6 under Put) may indicate rapid mobilization of sucrose for energy production or conversion into hexoses to support osmotic balance. In contrast, late-stage increases (e.g., day 13 in Put-treated plants) may result from enhanced photosynthetic C assimilation or reallocation of assimilates toward sucrose storage once stress responses are stabilized.

In addition to these C allocation dynamics, the variability in sucrose may also be linked to differential invertase activity under stress. Invertases, enzymes that regulate sucrose cleavage into glucose and fructose, and their activity is known to increase under salinity to facilitate osmolyte accumulation and respiration demand. Osmotic balance refers to the equilibrium of water and solutes across a membrane. Thus, reduced sucrose levels in early stages (e.g., day 6 under 100 mM NaCl + Put) may reflect enhanced vacuolar or cell wall invertase activity, driving hexose production to stabilize osmotic potential. Conversely, the late increase in sucrose (e.g., day 13 in Put-treated plants) could be associated with reduced invertase activity or enhanced sucrose resynthesis via sucrose-phosphate synthase, supporting assimilate storage and recovery processes.

This interpretation aligns with previous observations that Put treatment improved gas exchange (Section 3.2) and mitigated morphological damage (Section 3.1), possibly enabling better control of sucrose-invertase balance under stress. Overall, Put appears to modulate sugar allocation patterns in a dynamic, sugar-specific, and time-dependent manner.

### 3.4 Effects of put *s*pray on amino acids and polyamines content in leaves and roots

**Figure 4:**
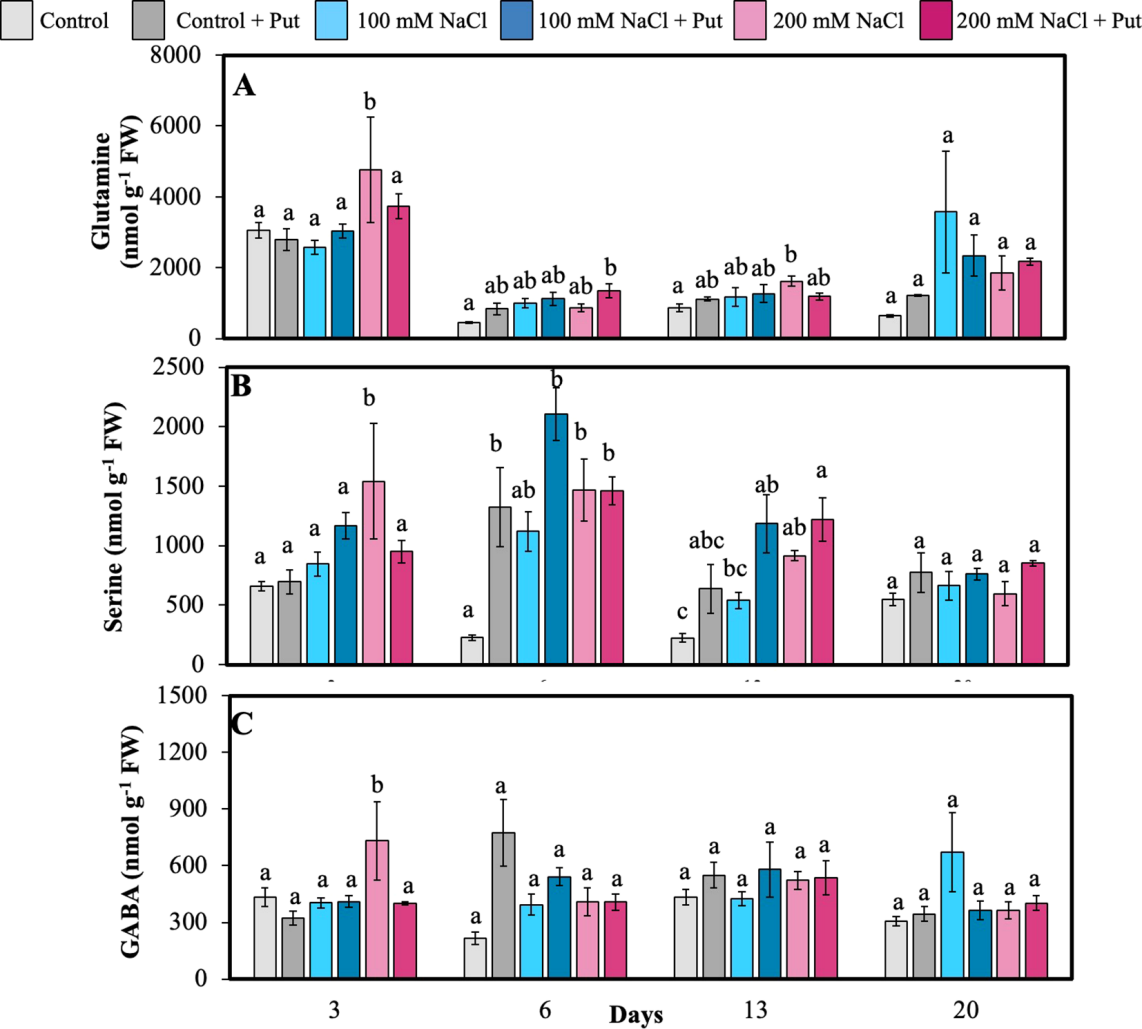
Effect of two different concentrations of NaCl (± Putrescine spray) on amino acids accumulation in hybrid poplar NM6 leaves over time. **(A)** Glutamine, **(B)** Serine, and **(C)** GABA measured at 3, 6, 13, and 20 days after salt treatment. Different letters indicate statistically significant differences (p< 0.05) among treatments. Data represent mean ± SE (n = 5).

**Figure 5:**
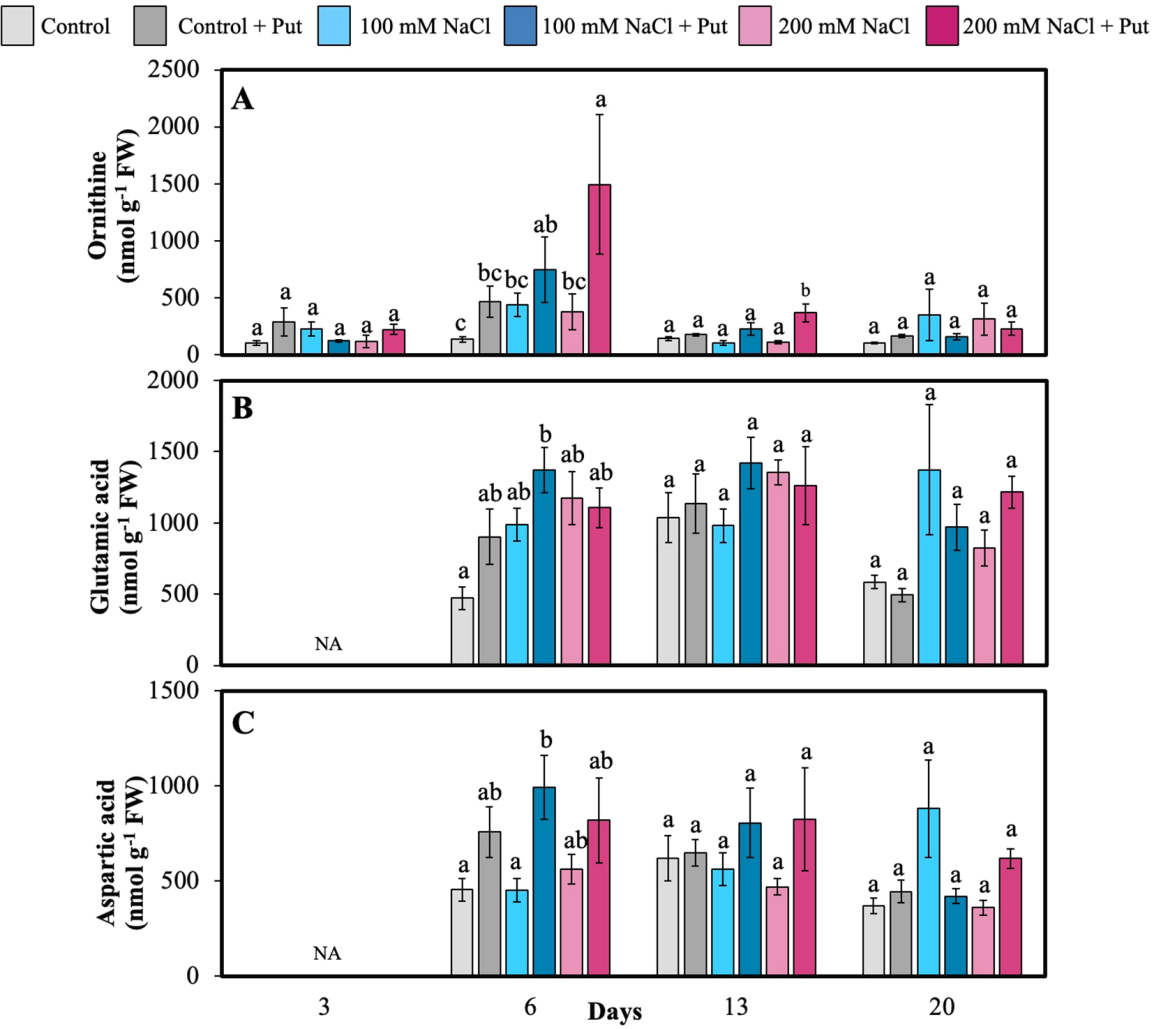
Effect of two different concentrations of NaCl (± Putrescine spray) on amino acids and polyamines accumulation in hybrid poplar NM6 leaves over time. **(A)** Ornithine levels, **(B)** Log Glutamic acid levels, and **(C)** Aspartic acid levels measured at 3, 6, 13, and 20 days after salt treatment. Different letters indicate statistically significant differences (p< 0.05) among treatments. NA indicates that the metabolite was below the detection limit at that timepoint. Data represent mean ± SE (n = 5).

AAs and PAs were measured in the 6th fully matured leaf on several days after NaCl treatment. Among all AAs, Arg + Thr + Gly and Gln were the most dominant. On day 3, 200 mM NaCl-treated plants had significantly higher levels of Gln, Ser, and GABA compared to all other treatments (**Figure 4**). Put-sprayed plants under 200 mM NaCl had significantly higher Gln, Ser, and Orn on day 6 (**Figures 4A, B**, **5A**). Elevated Gln and Orn may reflect increased N remobilization and precursor availability for polyamine biosynthesis, while higher Ser is often linked to photorespiratory adjustments under stress. Additionally, Put-sprayed plants under 100 mM NaCl showed significantly higher Glu and Asp compared to control plants on day 6 (**Figures 5B, C**), suggesting their possible role in energy metabolism and transamination reactions supporting stress acclimation. Ser was also significantly higher in Put-sprayed salt-treated plants on day 13, consistent with its function in 1-C metabolism and redox homeostasis.

**Figure 6:**
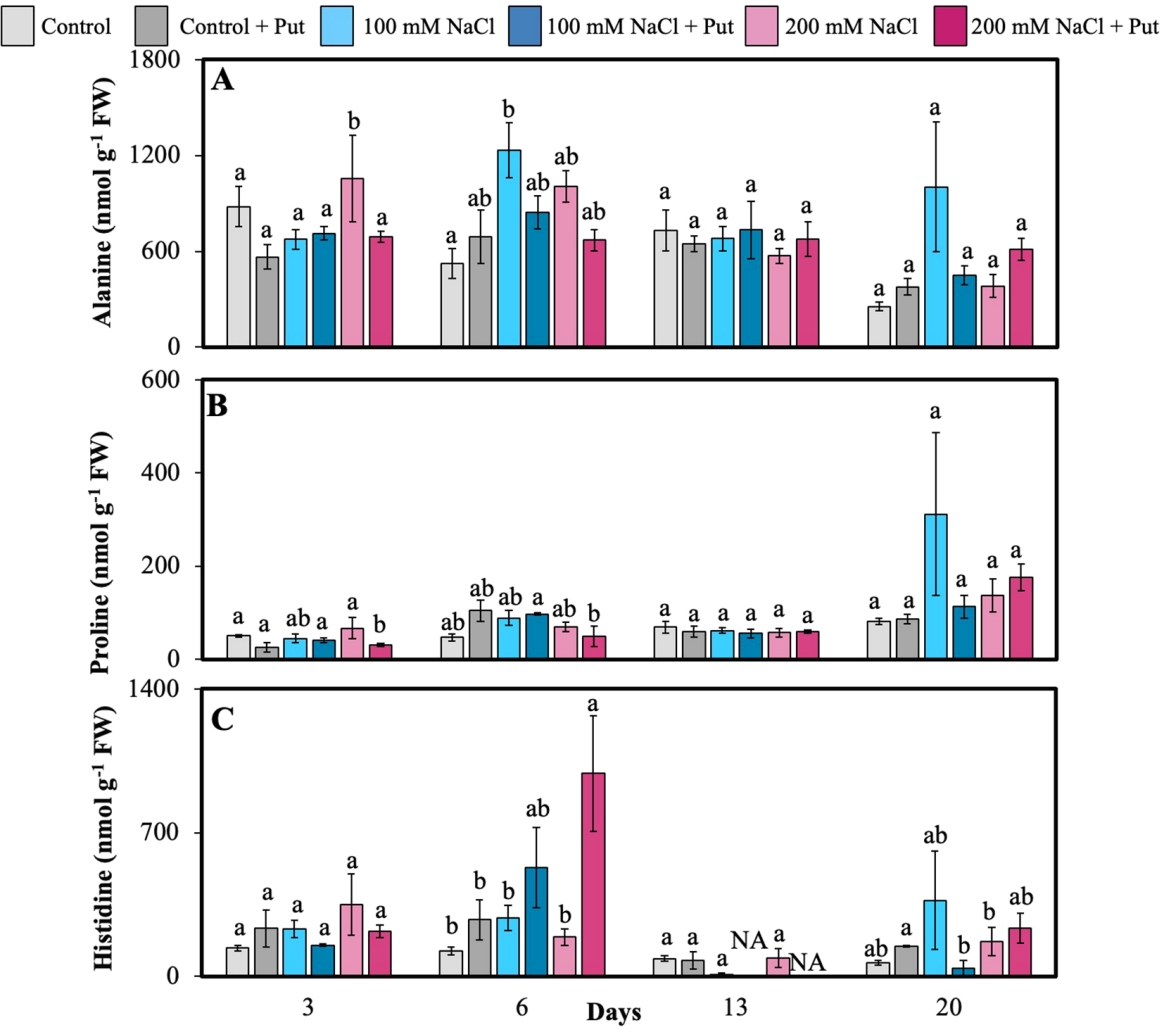
Effect of two different concentrations of NaCl (± Putrescine spray) on amino acids and polyamines accumulation in hybrid poplar NM6 leaves over time. **(A)** Alanine levels, **(B)** Proline levels, and **(C)** Histidine levels measured at 3, 6, 13, and 20 days after salt treatment. Different letters indicate statistically significant differences (p< 0.05) among treatments. NA indicates that the metabolite was below the detection limit at that timepoint. Data represent mean ± SE (n = 5).

Ala, Pro, Arg + Thr + Gly, Phe + Cys, and Ile were found to be significantly higher in unsprayed vs. sprayed 200 mM NaCl plants on day 3 (**Figures 6A, B**; **Supplementary Figures 2**, **3**). On day 6, Ala was also significantly higher in 100 mM NaCl plants compared to control, likely reflecting glycolytic overflow and C/N balance regulation under stress. Interestingly, Pro was significantly lower in Put-sprayed 200 mM NaCl plants on day 6 (**Figure 6B**), suggesting that Put application may reduce the reliance on Pro as an osmolyte, consistent with improved stress mitigation through alternative pathways. His was significantly higher in Put-sprayed 200 mM NaCl plants compared to other treatments on day 6 (**Figure 6C**), which may be linked to enhanced antioxidant potential and metal ion chelation under salinity.

**Figure 7:**
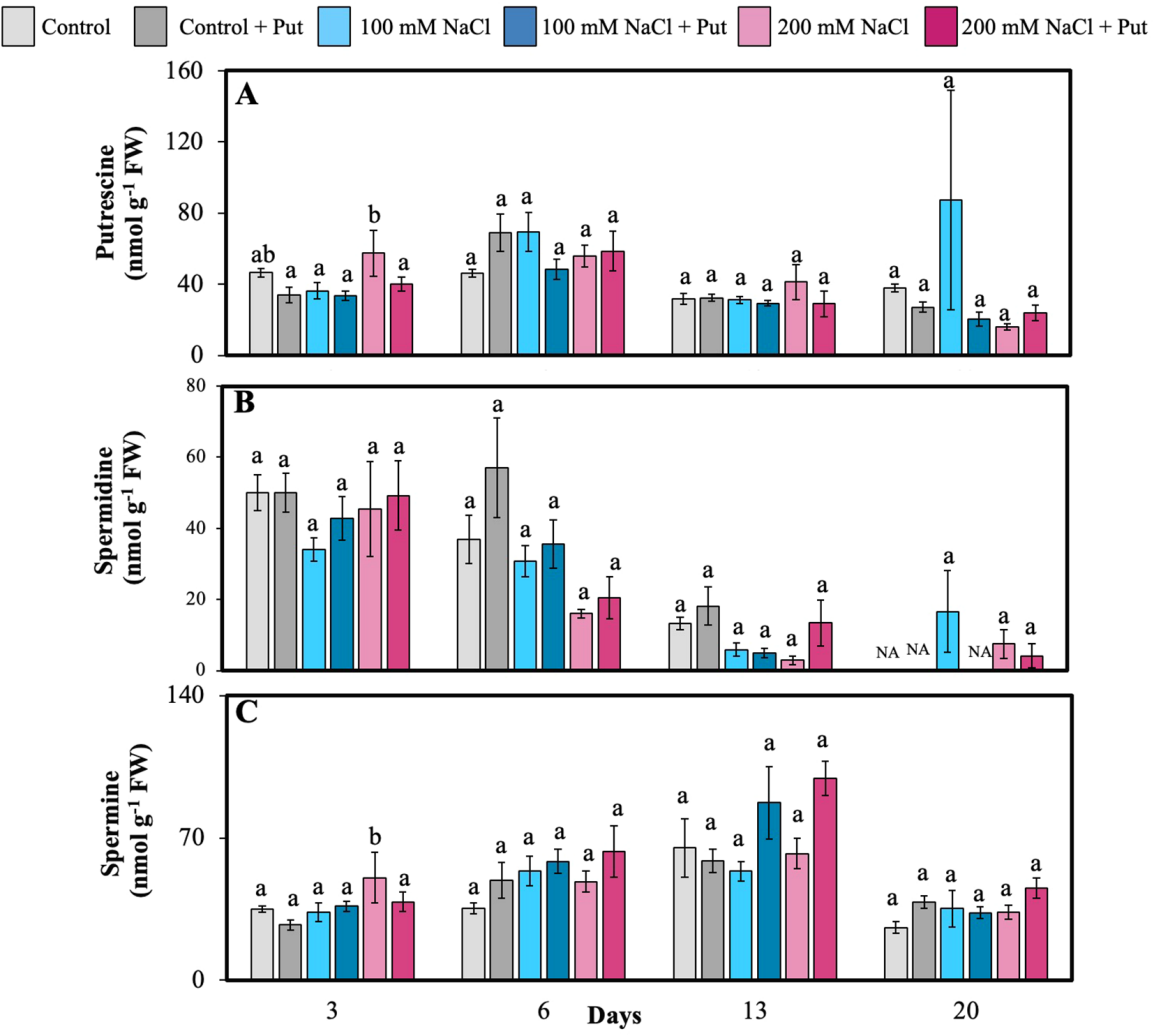
Effect of two different concentrations of NaCl (± Putrescine spray) on polyamines accumulation in hybrid poplar NM6 leaves over time. **(A)** Putrescine levels, **(B)** Spermidine levels, and **(C)** Spermine levels measured at 3, 6, 13, and 20 days after salt treatment. Different letters indicate statistically significant differences (p< 0.05) among treatments. NA indicates that the metabolite was below the detection limit at that timepoint. Data represent mean ± SE (n = 5).

Foliar spray of Put significantly altered endogenous Put and Spm content under 200 mM NaCl on day 3 (**Figure 7A**). However, PA levels varied under salt treatment on other days (**Figures 7B, C**). Since PAs are directly involved in stress signaling, membrane stabilization, and ROS scavenging, these changes suggest that Put supplementation plays a crucial role in modulating PA homeostasis to buffer salt-induced oxidative stress. This regulatory function of Put supplementation in PA homeostasis is a significant finding in the study.

**Figure 8:**
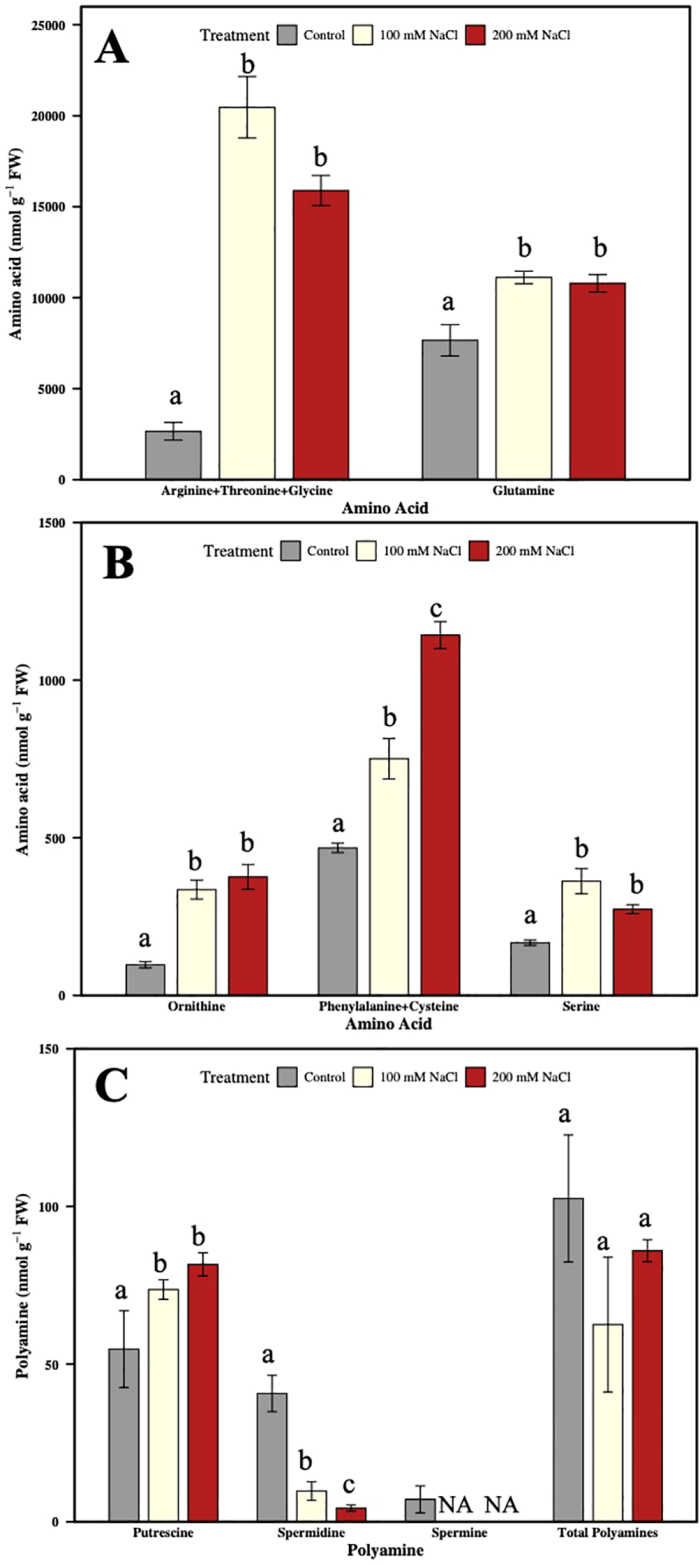
Effect of two different concentrations of NaCl on amino acids and polyamines accumulation in hybrid poplar NM6 roots. **(A, B)** Amino acids levels, **(C)** Polyamines levels measured at 21 days after salt treatment. Different letters indicate statistically significant differences (p< 0.05) among treatments. NA indicates that the metabolite was below the detection limit at that timepoint. Data represent mean ± SE (n = 4).

AAs and PAs in the roots were analyzed via HPLC only at 21 days after NaCl treatment, at the termination of the study. Salt significantly increased Arg + Thr + Gly, Gln, Orn, Phe + Cys, and Ser accumulation (in both concentrations of NaCl) compared to the control (**Figures 8A, B**). Accumulation of Arg and Orn is consistent with their role as key precursors for PA biosynthesis, while enhanced Ser and Gln point to sustained N remobilization under prolonged stress. Lys was significantly lower in 200 mM NaCl compared to control plants (**Supplementary Figure 4A**), which may indicate diversion of C/N fluxes away from Lys pathways under stress. Among PAs, Put was significantly increased under salt stress, while Spd was significantly reduced (**Figure 8C**), and Spm was undetected in salt-treated roots. This pattern reflects a shift in PA metabolism, where enhanced Put accumulation may support osmoprotection, while reductions in Spd/Spm highlight selective constraints on PA interconversion under salinity. Notably, total PA content in roots was lower in 100 mM NaCl-treated plants compared to both the control and 200 mM NaCl-treated plants, suggesting differential metabolic adjustment depending on stress severity.

Collectively, these results strongly confirms that salt stress reshapes AA and PA metabolism in a pathway-specific manner. At the same time, Put application modulates these shifts to promote nitrogen recycling, osmotic adjustment, and stress tolerance.

### 3.5 Combined analysis of polyamine metabolic cycle in roots

**Figure 9:**
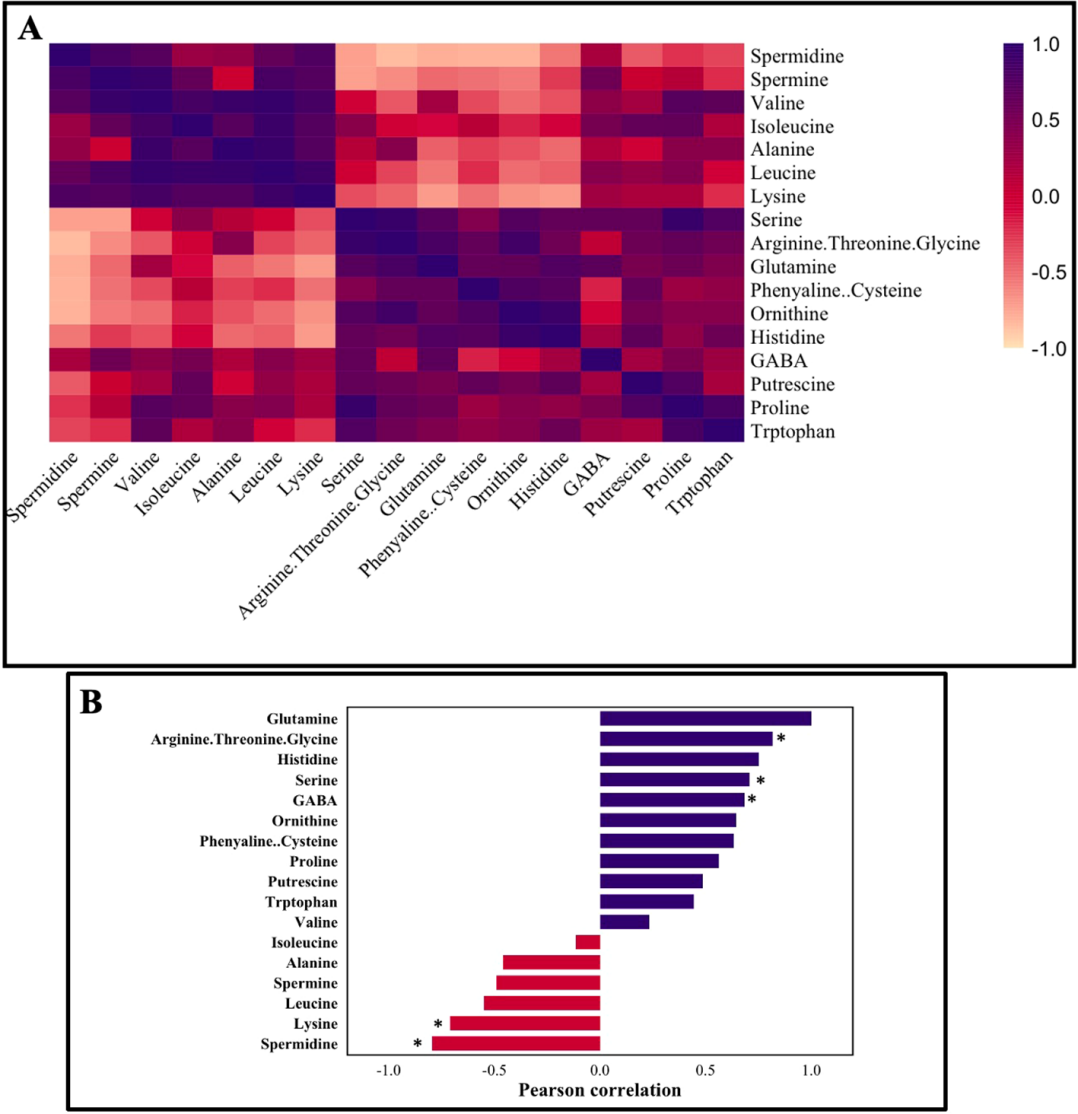
**(A)** Correlation coefficients of amino acids and polyamines analyzed in the poplar roots treated with two different concentrations of salt. **(B)** Pattern correlation analysis with Glutamine. The graph reports the significant features detected and ordered according to their correlation coefficient when correlated with Glutamine. The purple color represents a positive correlation, and the red color represents a negative correlation. Correlation distance- Pearson r. Correlation significant at *p< 0.05.

To assess the metabolic coordination among AAs and PAs in roots under salt stress, we conducted a Pearson correlation analysis based on their relative contents across control, 100 mM NaCl, and 200 mM NaCl treatments (**Figures 9A, B**). The heatmap revealed 2 distinct clusters: Cluster 1 included Gln, Pro, Ser, GABA, Put which showed strong positive correlations, several of which were statistically significant (e.g., Gln with Ser, GABA, Arg + Thr + Gly, p< 0.05). These metabolites are generally associated with stress adaptation and osmoprotection. Cluster 2 was composed of Spd, Spm, Ala, Lys, Leu, Ile, and Val. These showed negative correlations with Cluster 1 metabolites, most notably, Spd and Lys were significantly negatively correlated with Gln. This cluster represents more growth-associated or proteinogenic AAs and PAs.

The distinct separation of these clusters suggests that salt stress induces a shift in root N metabolism, favoring stress-responsive compounds (Cluster 1), while down regulating growth-linked metabolites (Cluster 2).

Mechanistically, these clusters indicate a coordinated rerouting of root N flux under salinity: the Gln–Orn/Put–GABA–Ser axis (Cluster 1) supports PA/GABA-shunt activity and feeds Cys–GSH production, strengthening ROS buffering and stress acclimation. In contrast, depletion of Spd/Spm and branched-chain/proteinogenic AAs (Cluster 2), and their negative correlations with Gln, suggest diversion of N away from protein synthesis and growth toward stress-protective metabolism. This pattern is consistent with a shift from growth to survival programs in salt-stressed roots.

## 4 Discussion

Soil salinity is a major global threat to agriculture and forestry, reducing plant growth through osmotic stress, ion toxicity, and oxidative damage (Hasanuzzaman et al., 2013; Dinneny, 2015; Safdar et al., 2019; Sun et al., 2022; Rady et al., 2023). Typical symptoms include leaf chlorosis and necrosis, stomatal closure, and reduced photosynthetic capacity (Hasanuzzaman et al., 2013; de Oliveira et al., 2013; Sun et al., 2022; Calhoun et al., 2023). While plants respond through physiological and biochemical reprogramming (Gupta and Huang, 2014; Yang and Guo, 2018; Raza et al., 2022; Wang et al., 2024a; Liu et al., 2025a), tolerance varies across species and genotypes.

PAs such as Put and Spd are widely recognized for their protective role against abiotic stress (Nahar et al., 2016; Xiong et al., 2018; Alcázar et al., 2020; Islam et al., 2021; Ma et al., 2022; Blázquez, 2024; Zhang et al., 2024), but studies in woody perennials remain limited. In the present study, we examined the effects of foliar Put application on NM6 under salt stress to evaluate its potential as a non-transgenic strategy for enhancing resilience. According to our hypothesis, Put application was expected to improve plant growth, photosynthesis, and metabolic stability under NaCl stress. Our results support this, showing significant increases in height and stem diameter, alleviation of leaf damage, and enhanced sugar accumulation, which together indicate a protective role of Put in salt-stressed NM6 poplars.

### 4.1 Effect of putrescine on physiological parameters

Salt stress induced visible symptoms in NM6 poplar by day 13, consistent with previous reports. Surprisingly, 200 mM NaCl-treated plants showed milder symptoms, which may reflect early acclimation or altered stress signaling. Salinity responses are biphasic: an early osmotic phase followed by a later ionic phase when leaf Na^+^ accumulates and accelerates senescence (Munns and Tester, 2008). A stronger salt signal (200 mM) likely provoked rapid ABA-mediated stomatal closure and growth arrest, lowering transpiration-driven Na^+^ delivery to leaves (Bharath et al., 2021; Hsu et al., 2021). This would allow earlier activation of Na^+^ exclusion/retrieval.

and vacuolar sequestration, for example via HKT-mediated xylem retrieval and NHX-type antiporters, thereby delaying visible ionic damage (Almeida et al., 2013; An et al., 2017; Bharath et al., 2021). By contrast, 100 mM allows sustained transpiration and leaf expansion, promoting progressive Na^+^ build-up and visible chlorosis/necrosis by this time point; additionally, salinity accelerates leaf senescence/abscission, shaping canopy appearance (Sade et al., 2018). Thus, milder symptoms at 200 mM NaCl reflect a more rapid protective response rather than reduced stress severity. Put-treated plants control plants developed minor tip necrosis but retained darker green foliage. Under combined stress (Put + NaCl), leaf damage shifted to central lamina, suggesting dose or interaction-dependent effects. Growth inhibition and wilting under salt aligned with responses seen in other species, such as cotton and poplar (Cao et al., 2020; Calhoun et al., 2023; Hu et al., 2023; Zhao et al., 2024a). Put application at 100 mM NaCl enhanced growth, supporting its role as a protective agent under moderate stress. Previous work shows that abiotic stress can induce long-term physiological adaptation, often described as stress memory (Wang et al., 2014; Georgii et al., 2019; Tikhomirova et al., 2022; Liu et al., 2022). Wang et al. (2025) reported that in *Populus alba × glandulosa*, photochemical activity improved in later stages of salt exposure. Consistently, our study showed that P_n_ in NM6 poplar improved by day 35 under salt stress, relative to earlier time points. Put-treated plants under salt stress also maintained higher g_s_ and E by day 35, similar to trends reported in *C. sinensis* and *C. sativus* (Xiong et al., 2018; Shen et al., 2019). These findings suggest that Put may enhance physiological resilience over time by modulating long-term stress responses and photosynthetic recovery. Total soluble protein declined under salt stress, likely due to increased degradation or inhibited synthesis, potentially contributing to elevated free amino acid levels. Put application under 100 mM NaCl restored protein content, but this effect was absent at 200 mM. No changes were observed in non-stressed plants, indicating the impact of Put is stress dependent. RWC slightly increased by day 13 under salt treatment, but was unaffected by Put, suggesting that protein fluctuations were not water-driven. FW/DW ratios also rose under salt, possibly reflecting osmotic adjustment.

### 4.2 Effect of putrescine on soluble sugars

Liang et al. (2018); Liu et al. (2022) and Zhang et al. (2024) reported that accumulation of soluble sugars is a common adaptive response to abiotic stress in plants, as reported in *Arabidopsis thaliana*, *Populus euphratica*, *Morus multicaulis* and *Oryza sativa*. Such osmotic adjustment, where sugars functions as key metabolite is well-documented in species like Tartary buckwheat. This trend was reflected in our observations, where early salt exposure corresponded with increased fructose and sucrose levels. The early and pronounced increase in fructose under severe salt stress may indicate an osmoprotective role. At the same time, Put application appeared to further enhance fructose accumulation even under moderate stress, possibly supporting better stress acclimation. Beyond osmotic roles, these shifts indicate active C partitioning: the early rise in fructose together with reduced glucose + galactose is consistent with invertase-mediated sucrose cleavage and rapid hexose phosphorylation/consumption, channeling C to respiration and compatible-solute synthesis while transiently reducing the free hexose pool. Reduction in glucose + galactose suggests hexose depletion under high salinity, could likely be due to increased utilization for respiration or metabolic adjustments. Put treatment maintained relatively higher levels, indicating a protective effect on hexose stability under salt stress. Later, the recovery of sucrose, especially with Put points to increased sucrose-phosphate synthase (SPS) activity and restored source capacity/phloem export, signaling a shift from catabolic to anabolic partitioning once defenses stabilize.

These shifts may be part of a stress-induced self-regulatory mechanism, wherein sugars contribute to osmotic balance, protect membrane stability, and support energy supply for metabolic adaptation. It has been previously established that such osmolyte accumulation also aids in maintaining high intracellular K^+^ and favorable Na^+^/K^+^ ratios, while preventing dehydration and oxidative damage (Liu et al., 2022; Azeem et al., 2023). Put application further enhanced sugar accumulation under salt stress, indicating its role in modulating C metabolism. This aligns with earlier findings in *Cucumis sativus* by Yuan et al. (2015), where PAs influenced sugar pathways, likely via PA-C metabolism crosstalk. Together, these results suggest that Put enhances sugar-mediated salt tolerance in poplar not only by promoting osmotic adjustment, but also by retuning C partitioning to sustain energy supply during the acute phase and to rebuild sucrose pools and sink support during acclimation.

### 4.3 Effect of putrescine on amino acids and polyamines

PAs are small N rich compounds known to mitigate salt-induced damage via multiple biochemical and physiological mechanisms (Alcázar et al., 2010; Minocha et al., 2014; Saha et al., 2015; Choudhary et al., 2023). Critically, their function extends beyond osmoprotection to redox control: PAs can directly scavenge ROS, stabilize membranes and ion homeostasis, and prime antioxidant defenses (e.g., SOD, CAT, APX, GR; components of the AsA-GSH cycle). In addition, PA oxidases (PAO/DAO) generate H_2_O_2_ as a controlled signal that upregulates stress-responsive genes and fortifies antioxidant capacity (Minocha et al., 2014; Alcázar et al., 2020).

We observed time-dependent changes in Put and Spm in leaves under 100 mM NaCl, with Put increasing and Spd declining over time; in roots, Put likewise increased under salt, while Spd declined and Spm was undetectable. This shift toward Put dominance is consistent with a PA profile that supports rapid ROS buffering and signaling during acclimation, while species- and tissue-specific declines in Spd/Spm reflect selective constraints on PA interconversion under salinity (Zhang et al., 2020b; Huo et al., 2020; Baniasadi et al., 2018; Ghalati et al., 2020; Shu et al., 2015). Marco et al. (2019) reported Arabidopsis Spm synthase mutants were more salt-sensitive, which further supports the importance of Spm in stress adaptation. Our data indicate that exogenous Put can still enhance tolerance by bolstering the PA–ROS interface.

PA catabolism is tightly linked to AA metabolism via shared intermediated and signaling molecules (Kundu, 2023; Blázquez, 2024). Consistent with this, salt exposure triggered early increases in Glu, Gln, GABA, and Pro in leaves, and Gln, Pro, Orn in roots, with Put spray further modulating these trajectories. Beyond osmo-protection, Pro and GABA contribute to redox homeostasis: Pro, via the Pro/P5C cycle, buffers the cellular NAD(P)H pool and can directly quench ROS; GABA, via the GABA shunt, feeds succinate into mitochondria and supports redox balance. Moreover, Put catabolism yields 4-aminobutanal, which is converted to GABA, mechanistically linking PA turnover to GABA accumulation and ROS management under salt (Gupta et al., 2013; Marco et al., 2019; Singh and Roychoudhury, 2020; Srivastava et al., 2021; Shao et al., 2022; Verslues et al., 2023).

Salt exposure triggered early increases in Glu, Gln, GABA, and Pro, indicating time-dependent reprogramming of N metabolism. Put spray further reshaped these trajectories, consistent with PA-AA crosstalk under salinity. In roots, salt stress promoted Gln, Pro, and Orn, pointing to diversion of N toward osmoprotection, PA precursor supply and redox buffering (Srivastava et al., 2021; Verslues et al., 2023; Wilhelmi et al., 2025). Beyond osmo-protection, Pro and GABA support mitochondrial redox balance, while the observed Gln-GABA-Gly-Ser positive correlations suggest that enhanced Gln assimilation fuels synthesis of these AAs and feeds the PA-GABA-GSH network. The decline in Lys under 200 mM NaCl may reflect its translocation to shoots, as observed in *C. sinensis* (Hijaz et al., 2018). Gln was positively correlated with GABA, Gly, and Ser, suggesting that increased Gln biosynthesis under salt stress may support synthesis of other AAs. Notably, the interaction between AA metabolism and the TCA cycle may contribute to enhanced salt tolerance, as Ali et al. (2019) suggested, providing a metabolic basis for the coordinated changes observed in our study. Taken together, our data support a model in which foliar Put strengthens salt tolerance by coupling osmotic adjustment with proactive ROS scavenging and signaling via the integrated PA–Pro–GABA–GSH network, providing a practical, low-cost strategy for hybrid poplar in saline-prone landscapes.

This study was conducted under controlled greenhouse conditions in a single growing season (2021), which enabled precise manipulation of salt and Put treatments while minimizing environmental variability. The consistency of treatment effects across multiple morphological, physiological, and biochemical measurements spanning two tissue types and several sampling timepoints provides robust internal validation of our findings. The mechanistic insights gained here establish a foundation for understanding Put-mediated salt stress tolerance in hybrid poplar. Future multi-year field trials under variable environmental conditions would be valuable for evaluating the agronomic potential and stability of putrescine applications in commercial poplar production systems, particularly for assessing long-term growth responses and economic feasibility in saline-affected landscapes.

## 5 Conclusions

This study demonstrates that *Populus nigra × maximowiczii* (clone NM6) exhibits adaptive physiological and metabolic responses under salt stress, particularly when treated with exogenous Put. At 100 mM NaCl, representing moderate salinity, Put-treated plants exhibited increased growth, conductivity, and transpiration rates and notable shifts in soluble protein, sugars, and AAs. These changes suggest enhanced osmotic regulation and metabolic stability, contributing to higher salt stress tolerance in NM6 poplar.

Given these responses under moderate salinity, NM6 shows potential as a viable species for cultivation on marginally saline land, mainly when supported by low-cost, non-transgenic treatments such as foliar Put application. Due to the unique role of deep-rooting systems, rapid growth, and soil-binding capacity, they further support their use in land restoration, sustainable silviculture, and bioenergy production.

The present findings derive from controlled greenhouse conditions and a short-term time frame; long-term, multi-season field validation is needed to confirm durability and operational feasibility. Our biochemical scope was also limited: we did not directly quantify Na^+^/K^+^ ionomics, ROS/antioxidant status, or hormone profiles (e.g., ABA/ethylene), and key enzymes underlying our interpretations (e.g., invertase isoforms, sucrose-phosphate synthase, PAO/DAO) were not assayed.

With the advent of newer technologies, future studies could incorporate transcriptomic and metabolomic analyses to map the full spectrum of PA-mediated regulatory pathways and their crosstalk with C and N metabolism. Integration with ionomics, targeted enzyme activity assays, hormone profiling, and antioxidant metrics may help mechanistically resolve the PA–AA network suggested here. Time-resolved leaf root sampling, field trials across soil salinity gradients, and AI-assisted multi-omics integration alongside conventional breeding could accelerate the development of stress-resilient NM6 for future saline-land management.

## Data Availability

The raw data supporting the conclusions of this article will be made available by the authors, without undue reservation.

## Supplementary material

Supplementary Figure 1 Workflow for the experimental design. This figure has been generated in Biorender.com.

Supplementary Figure 2 Effect of two different concentrations of NaCl (± Putrescine spray) on amino acids and photosynthesis accumulation in hybrid poplar NM6 leaves over time. **(A)** Arginine+Threonine+Glycine levels, **(B)** Leucine levels, and **(C)** Photosynthesis levels measured at several days after salt treatment. Different letters indicate statistically significant differences (p< 0.05) among treatments. NA indicates that the metabolite was below the detection limit at that timepoint. Data represent mean ± SE (n = 5).

Supplementary Figure 3 Effect of two different concentrations of NaCl (± Putrescine spray) on amino acids and polyamines accumulation in hybrid poplar NM6 leaves over time. **(A)** Valine levels, and **(B)** Isoleucine levels, and **(C)** Phenylalanine+Cystine levels measured at several days after salt treatment. Different letters indicate statistically significant differences (p< 0.05) among treatments. NA indicates that the metabolite was below the detection limit at that timepoint. Data represent mean ± SE (n = 5).

Supplementary Figure 4 Effect of two different concentrations of NaCl (± Putrescine spray) on amino acids and polyamines accumulation in hybrid poplar NM6 leaves over time. **(A)** Tryptophan levels, **(B)** Lysine levels, and **(C)** Methionine levels measured at several days after salt treatment. Different letters indicate statistically significant differences (p< 0.05) among treatments. NA indicates that the metabolite was below the detection limit at that timepoint. Data represent mean ± SE (n = 5).

Supplementary Figure 5 Effect of NaCl on amino acid accumulation in hybrid poplar NM6 roots and leaves. (A) GABA, Lysine, and Proline levels and (B) Isoleucine, Tryptophan, and Valine levels in roots measured on day 21 after salt treatment with control, 100 mM NaCl, or 200 mM NaCl. (C) Total amino acid levels in leaves measured at 3, 6, 13, and 20 days after treatment with control, 100 mM NaCl, 100 mM NaCl + Putrescine, 200 mM NaCl, or 200 mM NaCl + Putrescine. Different letters indicate statistically significant differences (p< 0.05) among treatments. NA indicates that the metabolite was below the detection limit at that timepoint. Data represent mean ± SE (n = 4) for **(A, B)** and (n =5) for **(C)**.
