# Supplementary figures and images for: Exogenous putrescine enhances salt tolerance in Populus nigra × maximowiczii: growth, physiological, and biochemical responses
Source: Front Plant Sci. 2026 Feb 27;16:1641288. doi: 10.3389/fpls.2025.1641288 (PMC12982397; doi:10.3389/fpls.2025.1641288)

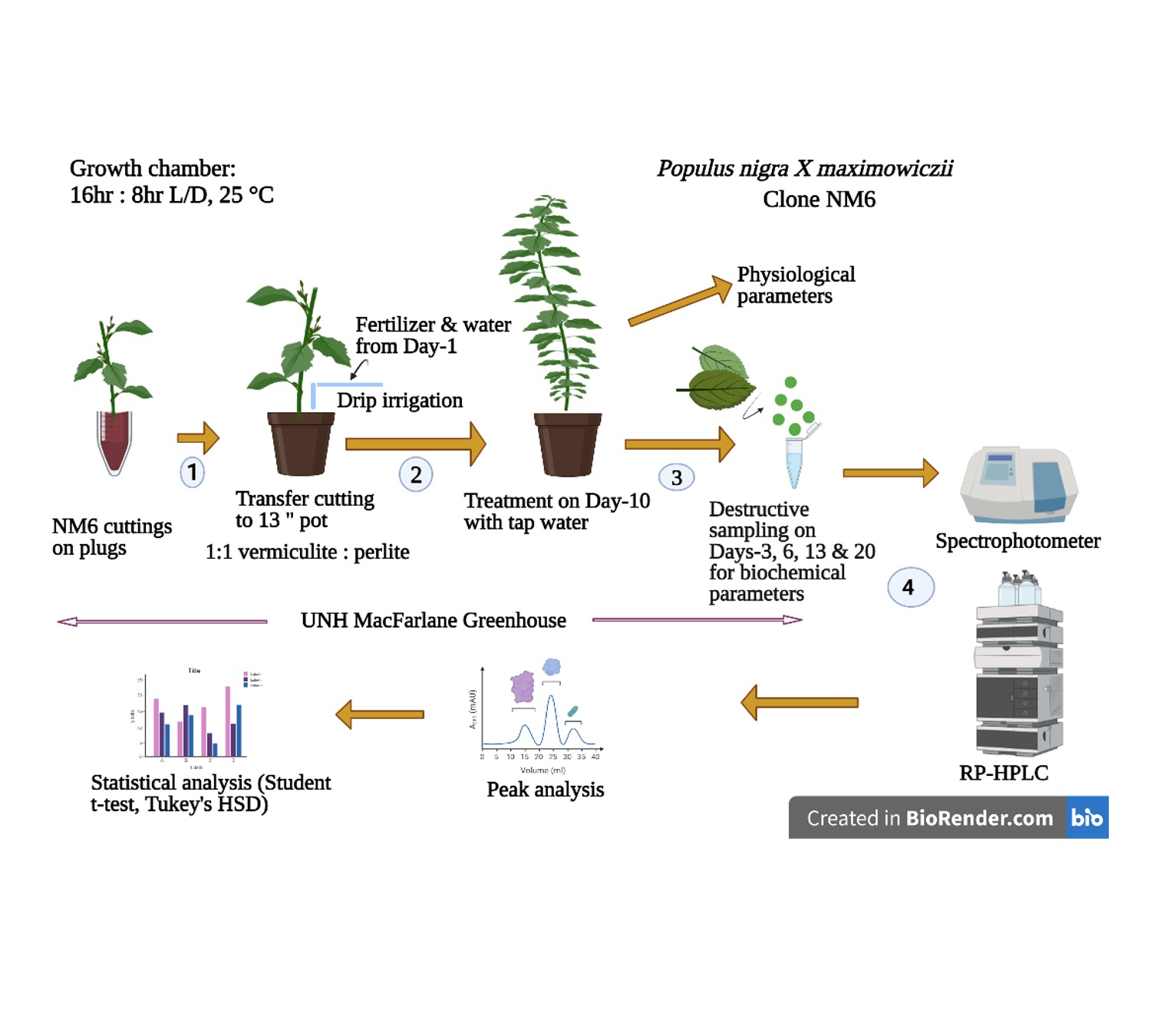

Supplement: Supplementary Figure 1 — Workflow for the experimental design. This figure has been generated in Biorender.com. [file Image1.jpeg]

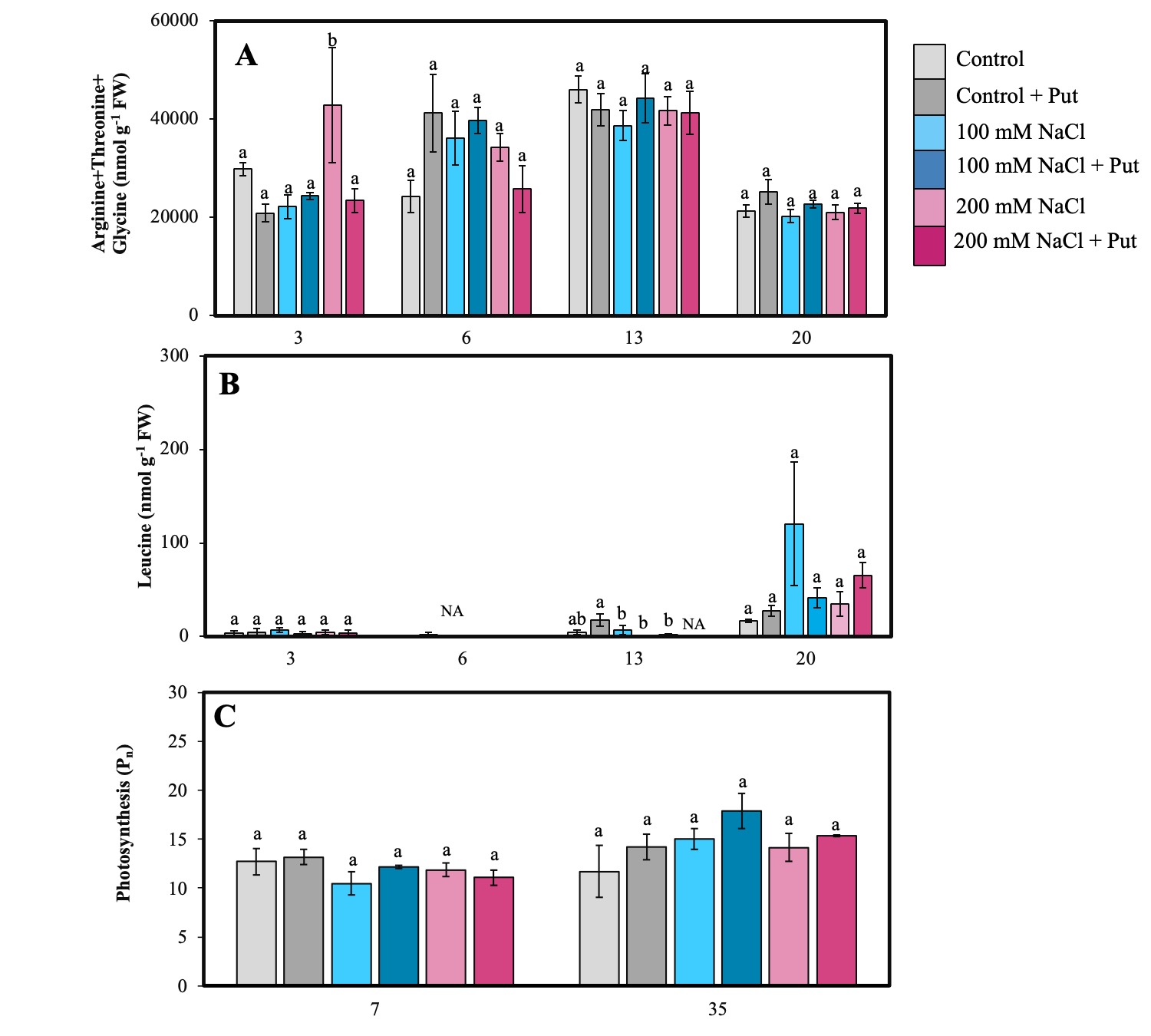

Supplement: Supplementary Figure 2 — Effect of two different concentrations of NaCl (± Putrescine spray) on amino acids and photosynthesis accumulation in hybrid poplar NM6 leaves over time. (A) Arginine+Threonine+Glycine levels, (B) Leucine levels, and (C) Photosynthesis levels measured at several days after salt treatment. Different letters indicate statistically significant differences (p< 0.05) among treatments. NA indicates that the metabolite was below the detection limit at that timepoint. Data represent mean ± SE (n = 5). [file Image2.jpeg]

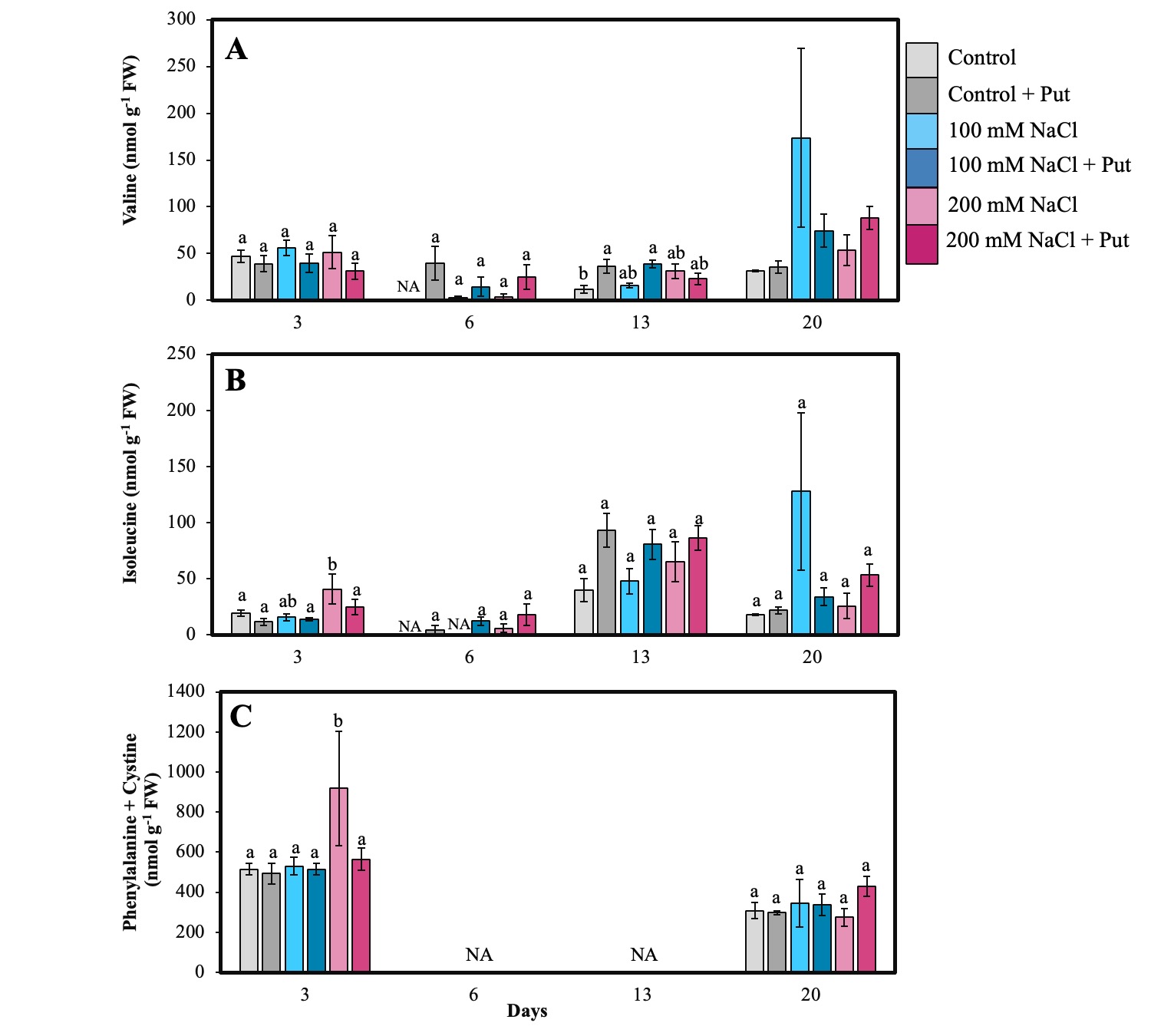

Supplement: Supplementary Figure 3 — Effect of two different concentrations of NaCl (± Putrescine spray) on amino acids and polyamines accumulation in hybrid poplar NM6 leaves over time. (A) Valine levels, and (B) Isoleucine levels, and (C) Phenylalanine+Cystine levels measured at several days after salt treatment. Different letters indicate statistically significant differences (p< 0.05) among treatments. NA indicates that the metabolite was below the detection limit at that timepoint. Data represent mean ± SE (n = 5). [file Image3.jpeg]

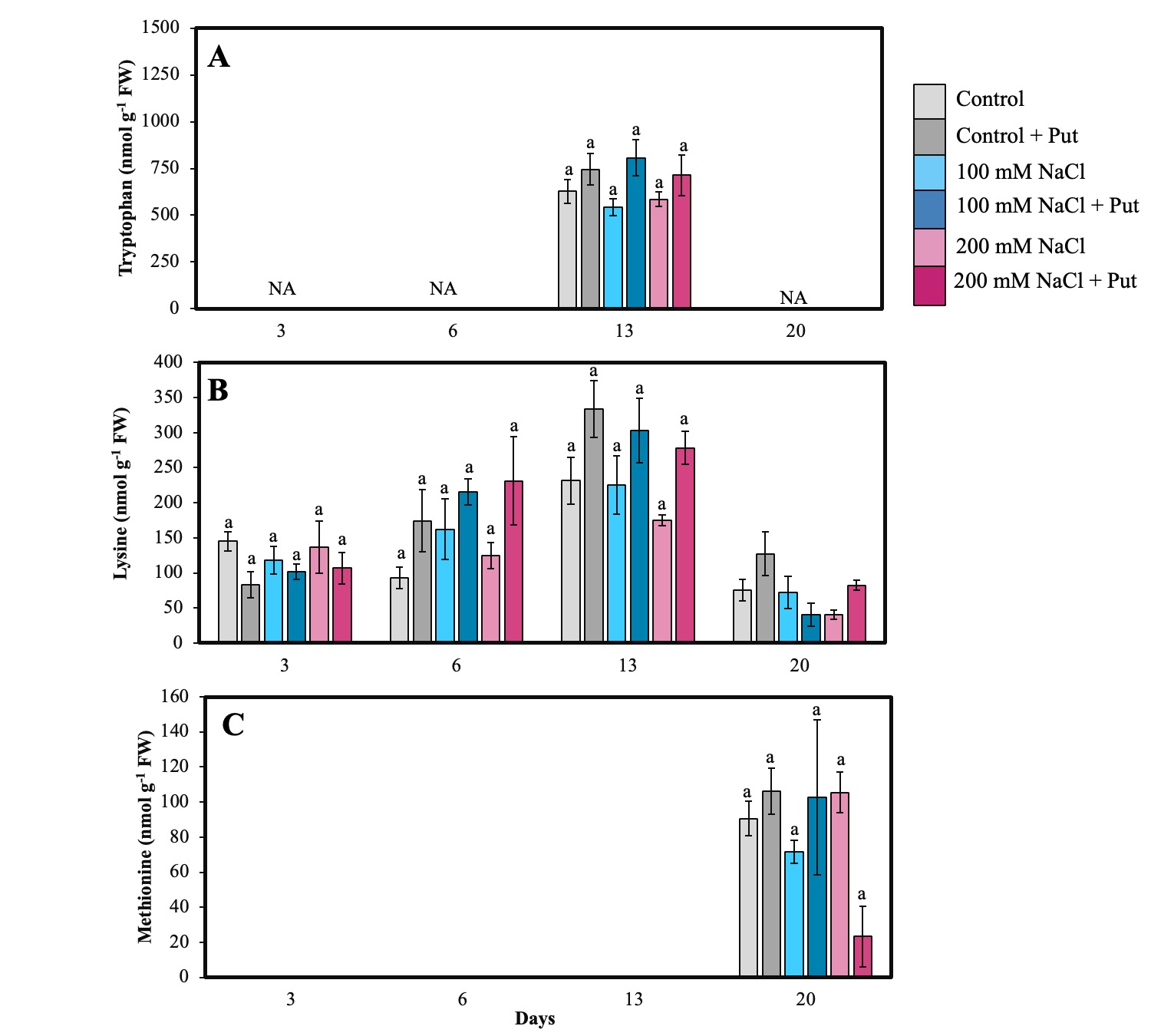

Supplement: Supplementary Figure 4 — Effect of two different concentrations of NaCl (± Putrescine spray) on amino acids and polyamines accumulation in hybrid poplar NM6 leaves over time. (A) Tryptophan levels, (B) Lysine levels, and (C) Methionine levels measured at several days after salt treatment. Different letters indicate statistically significant differences (p< 0.05) among treatments. NA indicates that the metabolite was below the detection limit at that timepoint. Data represent mean ± SE (n = 5). [file Image4.jpeg]

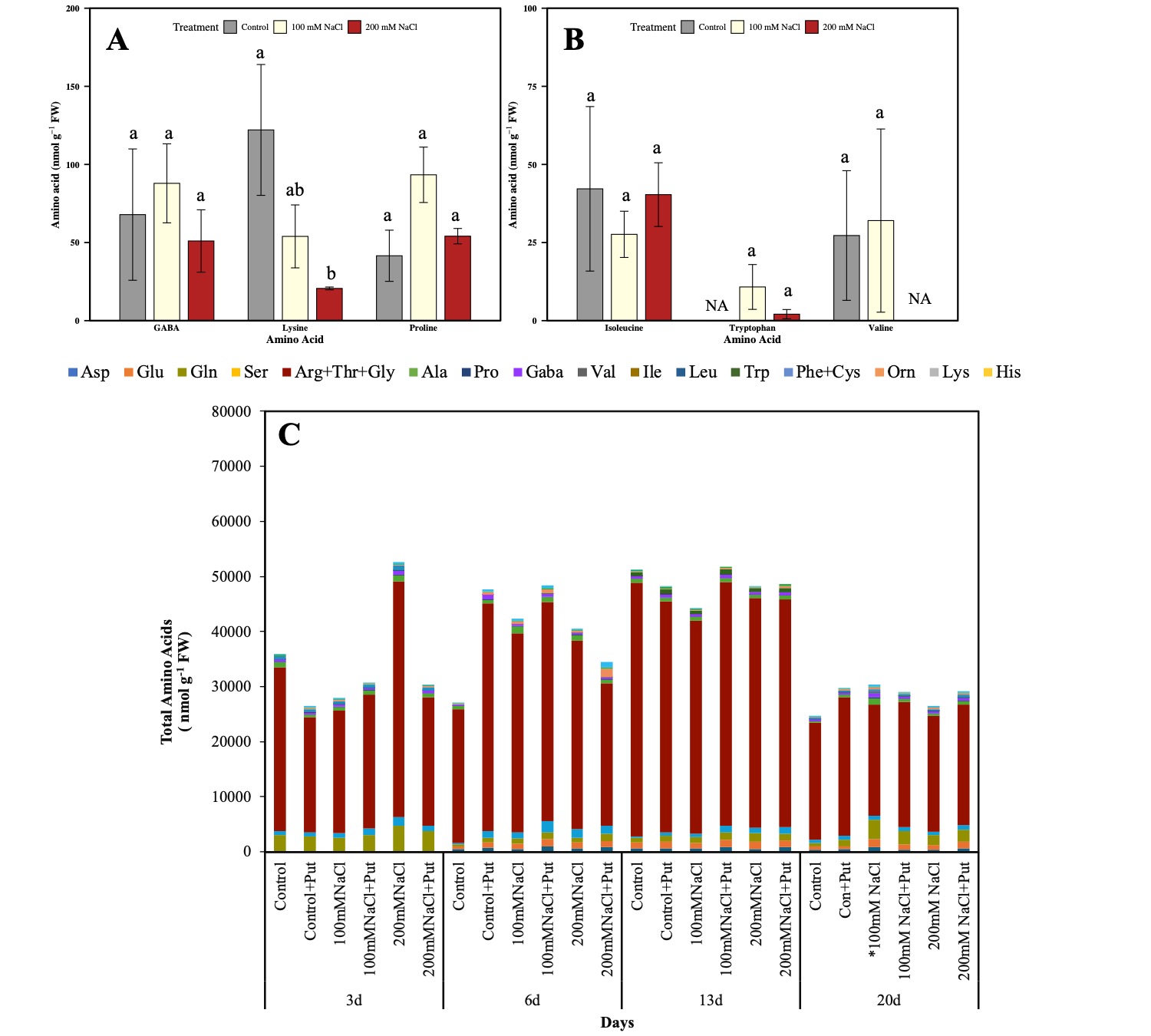

Supplement: Supplementary Figure 5 — Effect of NaCl on amino acid accumulation in hybrid poplar NM6 roots and leaves. (A) GABA, Lysine, and Proline levels and (B) Isoleucine, Tryptophan, and Valine levels in roots measured on day 21 after salt treatment with control, 100 mM NaCl, or 200 mM NaCl. (C) Total amino acid levels in leaves measured at 3, 6, 13, and 20 days after treatment with control, 100 mM NaCl, 100 mM NaCl + Putrescine, 200 mM NaCl, or 200 mM NaCl + Putrescine. Different letters indicate statistically significant differences (p< 0.05) among treatments. NA indicates that the metabolite was below the detection limit at that timepoint. Data represent mean ± SE (n = 4) for (A, B) and (n =5) for (C). [file Image5.jpeg]
